# Vancomycin heteroresistance among methicillin-resistant clinical isolates *S.* *haemolyticus*, *S.* *hominis*, *S.* *simulans*, and *S.* *warneri*

**DOI:** 10.1007/s42770-022-00870-7

**Published:** 2022-11-14

**Authors:** Magdalena Szemraj, Paweł Lisiecki, Paulina Glajzner, Eligia M. Szewczyk

**Affiliations:** Department of Pharmaceutical Microbiology and Microbiological Diagnostic, Medical University of Lodz, Muszyńskiego 1, 90-235 Łódź, Poland

**Keywords:** Daptomycin, Heteroresistance, Methicillin resistance, Vancomycin, *Staphylococcus*

## Abstract

Besides being an essential part of the skin microbiome, coagulase-negative staphylococci are the etiological factors of serious infections. The aim of the study was to evaluate the heteroresistance to vancomycin and the potential antimicrobial efficacy of teicoplanin and daptomycin against the multiresistant strains of *S. haemolyticus*, *S. hominis*, *S. warneri*, and *S. simulans*. The study covered 80 clinical coagulase-negative staphylococci. Teicoplanin, vancomycin, and daptomycin MICs for the tested strains were determined according to EUCAST recommendation. The *vanA* and *vanB* genes were searched. The brain heart infusion screen agar method detected vancomycin heteroresistance. The population analysis profile method and analysis of autolytic activity were applied for the strains growing on BHI containing 4 mg/L vancomycin. Seven *S. haemolyticus*, two *S. hominis*, and two *S. warneri* strains presented a heterogeneous resistance to vancomycin. Their subpopulations were able to grow on a medium containing 4–12 mg/L of vancomycin. Monitoring heteroresistance to peptide antibiotics, which are often the last resort in staphylococcal infections, is essential due to the severe crisis in antibiotic therapy and the lack of alternatives to treat infections with multiresistant strains. Our work highlights the selection of resistant strains and the need for more careful use of peptide antibiotics.

## Introduction

The importance of coagulase-negative staphylococci (CoNS) in nosocomial infections is beyond doubt [1–3]. *Staphylococcus epidermidis* is the most studied and clinically problematic, but other species cannot be ignored. Much less information is available on *S.* *haemolyticus*, *S.* *hominis*, *S.* *warneri*, and *S.* *simulans*. Meanwhile, they can cause nosocomial bloodstream infections and bacteremia due to medical procedures. They are related to implant infections, including joint prostheses, peritonitis, skin and soft tissue infections, otitis, or urinary tract infections [1, 3–6]. Multiresistant CoNS strains may pose a severe challenge for clinicians [7]. Their selection is a consequence of the antibiotic pressure on the natural skin microbiome in the hospital environment. Such strains are reservoirs of antibiotic resistance. Cremniter et al. [8] demonstrated subclones with lower sensitivity to glycopeptides among multiresistant CoNS clones circulating in hospitals.

Methicillin-resistant coagulase-negative staphylococci (MRCoNS) are particularly dangerous. They are clinically resistant to all β-lactam antibiotics and are also often resistant to other classes of antibiotics, mainly to aminoglycosides, macrolides, and lincosamides [9]. In the treatment of severe infections caused by multiresistant MRCoNS, glycopeptides, mainly vancomycin, are the first-choice antibiotics. Teicoplanin is used less frequently, as in vitro highly variable susceptibility is observed in CoNS, mainly in *S.* *haemolyticus* [10, 11]. Daptomycin is currently indicated as an alternative to vancomycin in the treatment of methicillin-resistant *S. aureus* infections [12]. This antibiotic is also used primarily to treat complicated skin and soft tissue infections. It is also used in the case of bacteremia caused by methicillin-resistant coagulase-negative strains in cases of vancomycin therapy failure [13].

Glycopeptides have unique mechanisms of action. Vancomycin inhibits cell wall peptidoglycan synthesis by interacting with the transpeptidation reaction. It also inhibits peptidoglycan remodeling, disrupting autolytic activity [14]. The mechanism of action of teicoplanin differs from that of vancomycin. The additional hydrophobic teicoplanin substituent interacts with the double lipid layer of the cell membrane, increasing the antibacterial effect [15]. The complexity of these mechanisms makes it more difficult for bacteria to develop resistance to glycopeptides than to other antibiotics. However, in recent years, the resistance of gram-positive bacteria to vancomycin has become more and more frequent, which significantly reduces the effectiveness of treatment [8]. In staphylococci, such resistance has been associated with the transfer of *van* genes from enterococci. The phenomenon of hetero resistance is also indicated. In the patient’s organism, in the population of primary cells marked as sensitive through diagnostic tests, a group of initially few resistant cells appears [16]. There are structural changes in cells, including thickening of the cell wall and a tendency to form cell aggregates [7, 8, 17]. This phenomenon is significant for clinicians when they more and more often encounter difficulties in obtaining a therapeutic effect after using glycopeptides.

Daptomycin (a lipopeptide antibiotic) is a cyclic peptide with a structure similar to that of cationic antimicrobial peptides (CAMP) [13]. It works by damaging the bacterial cell membrane. Resistance to daptomycin is rare and is associated with spontaneous mutations in genes involved in the biosynthesis of bacterial cell membranes [18]. However, when this occurs, resistant strains are selected.

The aim of the study was to evaluate the heteroresistance to vancomycin and the potential antimicrobial efficacy of teicoplanin and daptomycin against the multiresistant strains of *S.* *haemolyticus*, *S.* *hominis*, *S.* *warneri*, and *S.* *simulans*, species less frequently isolated from patients than *S. epidermidis*.

## Materials and methods

### Tested strains

From CoNS belonging to *S.* *haemolyticus*, *S.* *hominis*, *S.* *simulans*, and *S.* *warneri* species, collected from the hospital microbiological diagnostic laboratory in Łódź between 2014 and 2016, there were selected strains of the greatest clinical significance for further studies. Mainly, there were chosen strains from patients from intensive care unit, isolated from blood. In the next order, there were taken strains from other hospital units, isolated also from other material than blood. Finally, the study included 80 clinical CoNS isolates, including 23 *S.* *haemolyticus*, 19 *S.* *hominis*, 18 *S.* *simulans*, and 20 *S.* *warneri*. All *S.* *hominis* isolates and six *S.* *haemolyticus* isolates were obtained from blood. Other *S.* *haemolyticus* and *S.* *simulans* isolates came from infected wounds, while *S.* *warneri* came from many different sites, including from the eye, peritoneum, wounds, and urethra. In all cases strains were confirmed as etiological factor of existing disease. Identification of strains and sensitivity to cefoxitin have been presented previously [19]. The isolates were identified both by means of the MALDI-TOF technique and genetic methods. Nineteen *S.* *haemolyticus* isolates, 19 *S.* *hominis* isolates, four *S.* *simulans*, and six *S.* *warneri* were methicillin-resistant.

### Susceptibility to teicoplanin, vancomycin, and daptomycin

The teicoplanin, vancomycin, and daptomycin MIC values for the test strains were determined by E-test according to the manufacturer’s instructions. Resistance to teicoplanin or vancomycin was defined when MIC > 4 mg/L. For strains with reduced susceptibility to antibiotics (MIC = 1–2 mg/L), the MIC was confirmed by the broth microdilution method according to the guidelines of the European Committee on Antimicrobial Susceptibility Testing (EUCAST) [20]. Dilutions of the antibiotics were tested at the following concentrations: 16, 8, 4, 2, 1, 0.5, 0.25, and 0.125 mg/L. *S.* *aureus* ATCC 29,213 was used as control. Resistance to daptomycin was determined by a MIC > 1 mg/L.

### Detection of vancomycin heteroresistance

Screening on vancomycin brain heart infusion agar (BHI) was used to detect heteroresistance to vancomycin in all strains tested according to Satola et al. method with modifications for CoNS [21, 22]. Suspensions at a density of the 0.5 McFarland standard were prepared from the overnight cultures of the strains on blood agar. Four 10 µL drops of each suspension were applied to a BHI agar plate (Oxoid) containing 4 mg/L vancomycin (Sigma). The plates were incubated at 37 °C for 48 h. After incubation, the grown colonies were counted. A strain was considered heterogeneous resistant coagulase-negative staphylococci (hVICoNS) when at least one drop contained at least two resistant colonies. Experiment was repeated three times for each individual strain.

### Population analysis profile (PAP) for vancomycin heteroresistance

Vancomycin heteroresistance in subpopulations was marked for the strains growing on BHI agar plate containing 4 mg/L vancomycin according to the method described earlier [23]. A serial tenfold dilution in sterile saline bacterial suspensions of 0.5 McFarland density was prepared. Subsequently, 100 μL was put on BHI agar plates containing vancomycin at concentrations 2, 4, 6, 8, 10, 12, 14, and 16 mg/L. Plates were incubated at 35 °C for 48 h. After this period, the number of colonies growing in the presence of each concentration of vancomycin was counted. Heterogenous vancomycin intermediate resistant *Staphylococcus aureus* (hVISA) strain Mu3 (ATCC 700698) was a positive control, while the vancomycin-susceptible *Staphylococcus aureus* (VSSA) strain ATCC 29213 was a negative control. Each strain was tested three times.

### Whole-cell autolysis assay

The autolytic activity induced by Triton X-100 was tested on subpopulations of strains that grew in vancomycin concentration ≥ 6 mg/L in the PAP test according to the method described by Hanaki et al. [24] with modifications (Nunes et al. [17]).

The strains were grown in BHI broth with vancomycin at half the corresponding MIC and incubated at 35 °C for 24 h. The bacterial cells were then washed with cold water and resuspended in 100 mL of 0.05 M Tris–HCl buffer (pH 7.2) with 0.05% Triton X-100 (Sigma). The initial optical density at 600 nm (OD600) was determined, and each suspension was incubated at 35 °C with shaking (250 rpm) for 4.5 h. After this period, another determination was made. These procedures were run at least three times.

### Detection of the *vanA* and *vanB* genes

For strains that had reduced susceptibility to vancomycin (MIC = 1–2 mg/L), the *vanA* and *vanB* genes were searched. Genomic DNA was isolated using the Genomic Micro AX Staphylococcus Gravity kit (A&A Biotechnology, Poland) according to the manufacturer’s protocol. The genes were detected using the previously described primers and parameters [25, 26]. DNA amplification was performed in a thermocycler (Biometra, Germany). The reaction products were identified by electrophoresis (70 V, 1.5 h) in 1% (w/v) agarose gels containing the dye Midori Green DNA (Nippon Genetics Europe, Germany). The sizes of the amplification products were identified by using the DraMix Marker or the DNA Marker 2 (A&A Biotechnology, Poland). *Enterococcus faecalis* NCTC 12201 (*vanA*) and *E. faecalis* ATCC 51299 (*vanB*) were used as controls.

### The statistical analysis

The associations between the results obtained for species vs. resistance to antibiotics prevalence were determined using the chi-squared test. *p* < 0.05 proved the significance of these relations. For statistical analysis, STATISTICA 13.1PL software was used (StatSoft 2016, Poland).

## Results

Of the 80 CoNS strains isolated primarily from blood and wound infections studied earlier, 49 isolates carried the *mecA* gene. They were phenotypically methicillin-resistant, indicating clinical resistance to β-lactams. Many of them were multiresistant. *S.* *hominis* and *S.* *haemolyticus* strains showed resistance to the greatest number of antibiotics, including all β-lactams, aminoglycosides, and ciprofloxacin. Multiresistance, including methicillin resistance, also affected *S.* *simulans* and *S.* *warneri* strains. All tested methicillin-susceptible strains of coagulase-negative staphylococci (MSCoNS) were simultaneously susceptible to all glycopeptides. Most of the methicillin-resistant strains in our collection were also clinically susceptible to vancomycin and teicoplanin. However, three *S.* *haemolyticus* and two *S.* *hominis* were resistant to teicoplanin. One strain of *S.* *hominis* had teicoplanin MIC = 32 mg/L.

**Fig. 1:**
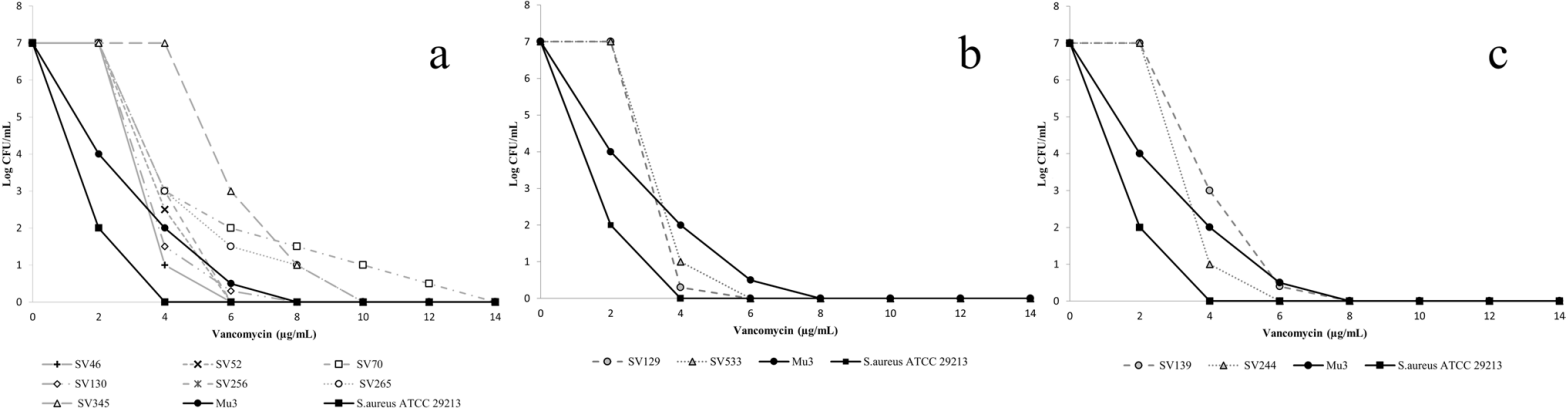
Population analysis profiles (PAPs) for seven of *S.* *haemolyticus*, two of *S.* *hominis*, and two of *S.* *warneri* with reduced susceptibility to vancomycin. **a** *S. haemolyticus*, **b** *S. hominis*, and **c** *S. warneri*

Following the EUCAST susceptibility criteria, which state that a strain should only be considered resistant to vancomycin when the MIC value is greater than 4 mg/L, all MRCoNS strains tested by us had to be classified as susceptible to this antibiotic. However, although most of them showed MIC values below 0.25 mg/L, the growth of the four *S.* *haemolyticus* strains was inhibited just above 2 mg/L. Three other strains of *S.* *haemolyticus*, two *S.* *hominis*, and two *S.* *warneri* had a vancomycin MIC of 1 mg/L. All *S.* *simulans* strains exhibited MICs of daptomycin ≤ 0.125 mg/L and vancomycin ≤ 0.25 mg/L. All tested strains were susceptible to daptomycin (MIC ≤ 0.25 mg/L). The results are presented in Table 1. Eleven strains (22% of all tested strains) that showed reduced susceptibility to vancomycin (MIC = 1–2 mg/L) were grown on a BHI agar plate with 4 mg/L of vancomycin. All of them had heterogeneous resistance profiles confirmed with PAP test. Their subpopulations could grow on a medium containing 4–12 mg/L of vancomycin. Three *S.* *haemolyticus* isolates, two *S.* *hominis* and one *S.* *warneri*, were grown in a 4 mg/L vancomycin medium. One *S.* *haemolyticus* isolate and one *S.* *warneri* isolate increased the subpopulation by 6 mg/L. The subpopulations of the three *S.* *haemolyticus* isolates grew at higher vancomycin concentrations. PAP test results for seven clinical strains of *S.* *haemolyticus*, two *S.* *hominis*, two *S.* *warneri*, and control strains (*S. aureus* ATCC 29213, susceptible to vancomycin and Mu3, hVISA) are shown in Fig. 1.

**Table 1:** Vancomycin and daptomycin MICs by E-test and species distribution MRCoNS

Species (*n*)	Teicoplanin MIC (mg/L)					Vancomycin MIC (mg/L)					Daptomycin MICs (mg/L)
	< 4	4	6	8	≥ 8		< 1	1	2	≥ 4	< 1
*S. haemolyticus* MS (4)	4	0	0	0	0		4	0	0	0	4
*S. haemolyticus* MR (19)	13	3	1	1	1		12	3	4	0	19
*S. hominis* MS (0)	0	0	0	0	0		0	0	0	0	0
*S. hominis* MR (19)	17	0	0	0	2		17	2	0	0	19
*S. simulans* MS (14)	14	0	0	0	0		14	0	0	0	14
*S. simulans* MR (4)	4	0	0	0	0		4	0	0	0	4
*S. warneri* MS (14)	14	0	0	0	0		2	0	0	0	14
*S. warneri* MR (6)	6	0	0	0	0		16	2	0	0	6

*n*, number of strains; *MS*, methicillin-susceptible; *MR*, methicillin-resistant; *MIC*, minimum inhibitory concentration

Whole cell autolysis tests were performed on five strains whose subpopulations grew at vancomycin concentrations ≥ 6 mg/L. They were *S.* *haemolyticus* isolate and *S.* *warneri* isolate (MIC = 6 mg/L), two *S.* *haemolyticus* MIC = 8 mg/L, and one *S.* *haemolyticus* MIC = 12 mg/L. Correspondingly, subpopulations growing at vancomycin concentrations of 6 and 8 mg/L showed higher autolysis rates than their parent strains. Among the resistant subpopulation of *S.* *haemolyticus* SV265, a strain exhibiting significant teicoplanin resistance (12 mg/L), autolysis was lower than the parent strain. The results are presented in Table 2. None of the tested strains carried the *vanA* and *vanB* genes.

**Table 2:** Characteristics of vancomycin-heteroresistant strains (according to PAP test)

Species	Strain	Isolation site	MR (CF disk inhibition zone diameters in mm)	Growth on BHI medium with vancomycin (mg/L)	MIC VAN (mg/L)	MIC TEIC (mg/L)	Autolytic activity (%)
*S. warneri*	SV139	Wound	+ (18)	6	1	3	13
	SV139V_6_						14
*S. haemolyticus*	SV130	Wound	+ (10)	6	2	4	10
	SV130V_6_						12
	SV70	Blood	+ (0)	8	2	4	12
	SV70V_8_						13
	SV345	Blood	+ (0)	8	1	4	12
	SV345V_8_						14
	SV265	Wound	+ (8)	12	1	12	12
	SV265V_12_						9

*MR*, methicillin resistance; *CF*, cefoxitin (30 µg); *MIC*, minimum inhibitory concentration; *VAN*, vancomycin; *TEIC*, teicoplanin; *Vx*, derivative strain selected in the presence of vancomycin in concentration _x_ (mg/L)

## Discussion

The occurrence of multiresistance in staphylococci, including methicillin resistance, is a growing problem in achieving the desired therapeutic effect in the treatment of infections. Therapeutic alternatives against strains resistant to β-lactam antibiotics are glycopeptides, which are usually effective. This applies to both *S. aureus* and CoNS infections, also those species which are rarely isolated from clinical materials. In this work, we have shown some alarming cases of selecting glycopeptide-resistant strains. We focused our research on four species: *S.* *hominis*, *S.* *haemolyticus*, *S.* *simulans*, and *S.* *warneri*, which, although constantly present on the skin of patients and undergoing selective pressure during antibiotic treatment, rarely cause severe infections on their own. Nevertheless, they pose a serious risk to immunocompromised patients. The strains we studied were isolated from such cases. We noticed an increase in vancomycin MIC values for some methicillin-resistant strains and decided to investigate the cause. Most studies show the problem of resistance to teicoplanin, vancomycin, and daptomycin in *S. epidermidis* and *S.* *haemolyticus*, while few data are available for other CoNS species [13, 17, 27].

Vancomycin is the drug of the first choice for the treatment of serious infections such as blood and soft tissue infections caused by MRCoNS. Increased use of glycopeptide antibiotics results in selection of strains with reduced sensitivity to them. *S. aureus* strains with reduced susceptibility to vancomycin (MIC = 4–8 mg/L) were reported first in 1997 in Japan and referred to as intermediate vancomycin-resistant *Staphylococcus aureus* (VISA) [28]. Currently, vancomycin resistance also applies to coagulase-negative species, especially those methicillin-resistant, which are important in nosocomial infections [27, 29]. Most of the methicillin-resistant strains in our collection of 80 strains were clinically susceptible to vancomycin and teicoplanin. However, seven strains of *S.* *haemolyticus*, two *S.* *hominis*, and two *S.* *warneri* showed reduced susceptibility to vancomycin and three *S.* *haemolyticus* and two *S.* *hominis* were resistant to teicoplanin.

Our *S.* *haemolyticus* strains grew in the presence of the highest vancomycin concentrations in the medium (6–12 mg/L). This ability may contribute to the generation of multiresistant strains of *S.* *haemolyticus* [30]. Nevertheless, according to EUCAST breakpoints, these strains would be considered clinically susceptible. Meanwhile, vancomycin treatment in such cases may be ineffective and may result in the selection of resistant clones.

Previous use of β-lactam antibiotics may lead to the selection of strains heterogeneously resistant to vancomycin. Roch et al. demonstrated in their studies that methicillin-resistant *S. aureus*, clinically susceptible to vancomycin, treated with three different β-lactams (imipenem, ceftazidime, and ceftriaxone), can select toward hVISA [31]. Methicillin-resistant *S. aureus* infections are common, e.g., in patients with burns, and require other therapeutic options [32].

Vancomycin resistance was originally thought to be a consequence of the transfer of the *vanA* operon encoded on the TnI546 transposon from enterococci. However, reports of such strains are rare [33]. hVISA strains have been reported with increasing frequency. Their phenotype is heterogeneous. Most of the cells in the culture are susceptible. However, the resistant part can grow in a medium with a vancomycin concentration ≥ 4 mg/L. The molecular basis of this phenotype is not exactly known. Many genes are believed to contribute to this [34]. None of the strains we tested had the *vanA* or *vanB* genes. The attempt to search for them in the DNA of colonies growing on the vancomycin medium also failed. This observation confirms the view that the lack of therapeutic effect is a consequence of reduced vancomycin susceptibility rather than complete resistance caused by the presence of the *van* complex genes [7, 8]. The acquisition of this resistance is associated with slight mutational changes in several other genes. It turns out that vancomycin resistance may depend on many genes [34]. Such resistance results from a disturbed activity of genes involved in the processes of cell wall synthesis, which could somehow be associated with resistance to β-lactam antibiotics. The reduced production of autolysins responsible for recycling the cell wall increases its thickness, which was shown in the electron microscope photos [17, 35, 36]. Vancomycin sensitivity impairment is also associated with dysfunction of the *agr* system leading to metabolic changes. McGuinnes et al. identified several genes that indirectly contribute to the vancomycin resistance phenotype. That explains why the ability to grow in the presence of high vancomycin concentrations is sometimes associated with other features found in resistant strains [34].

The method used in many studies to determine heteroresistance to glycopeptides in staphylococci is PAP [17, 22]. This resistance was demonstrated in the PAP test in eleven strains studied by us. That allows us to define them as heterogeneous CoNS with intermediate resistance to vancomycin. However, the mechanism of this resistance still needs elucidation. Demonstration of heteroresistance to vancomycin is impossible in a routine laboratory test. However, when the MIC glycopeptide values for the tested strain are 1–2 mg/L, it seems necessary to consult the laboratory with clinicians to determine whether the patient has already been treated with these antibiotics. Using them again poses a high risk of therapy failure. The fact that such a decrease in the MIC value should be seriously analyzed is also shown in the studies by Natoli et al. on strains obtained from patients of the transplant hematology department [29]. The studies of other researchers [17, 22] also indicate that heteroresistance more often concerned strains isolated from patients who had been previously treated with vancomycin. It seems necessary to develop glycopeptide sensitivity tests evaluating the heteroresistance of strains to this group of antibiotics.

It must be considered that the reduced vancomycin susceptibility in CoNS may occur through a mechanism other than that of *S. aureus*. That was described for *S. capitis* and *S. epidermidis* [37]. In the case of the strains we studied, reduced vancomycin sensitivity did not always mean resistance to teicoplanin and vice versa (strains of *S.* *warneri* and *S.* *hominis*). A decreased susceptibility of *S.* *warneri* to vancomycin has already been reported [17, 30], but *S.* *hominis*, unlike in our work, is usually considered sensitive [38]. Therefore, the strains selected in our study are a very interesting collection for future research on the mechanisms of glycopeptide resistance.

Due to the reduced susceptibility to vancomycin, it is necessary to use new antibiotics [39]. Daptomycin is recommended to treat bloodstream infections [40, 41]. In our study, all strains were sensitive to daptomycin. Similar results were presented by Sader and Jones [13]. They showed that despite the reduced vancomycin susceptibility observed in *S. epidermidis*, *S.* *haemolyticus*, and *S.* *xylosis* strains, these strains remained sensitive to daptomycin. The efficacy of daptomycin was also reported by Pereira et al. [42]. However, it should be emphasized that strains of *S.* *auricularis*, *S.* *warneri*, *S. capitis*, *S.* *saprophyticus*, *S.* *hominis*, and *S. epidermidis* species resistant to daptomycin have been already demonstrated [26].

In a 2019 study, an Australian group [37] showed that the mechanism of daptomycin resistance in CoNS depended not on alteration of cell surface charge like in *S. aureus* but on mutations in *walK* and *walR* genes, an essential two-component regulatory system controlling cell wall biogenesis. The transfer of the altered *walK* gene from *S. capitis* to *S. epidermidis* resulted in reduced susceptibility not only to daptomycin but also to vancomycin. The emerging resistance to peptide antibiotics may depend on various mechanisms.

It should be considered that antibiotic treatment may lead to the selection of resistant strains among pathogens also among species constantly present in the human skin microbiome and less often isolated from severe clinical cases. The search for resistant strains of less studied species should be continued. It is also necessary to study in detail the genetic mechanism leading to the phenotypic changes we have observed.

Monitoring heteroresistance to peptide antibiotics, which are often the last resort in staphylococcal infections, is essential due to the severe crisis in antibiotic therapy and the lack of alternatives to treating infections with multiresistant strains. Our work is part of the call for more prudent use of antibiotics, with particular emphasis on peptide antibiotics.

## Data Availability

The datasets used and/or analyzed during the current study are available from the corresponding author on reasonable request.
